# Evaluation of the effect of vascularized fibula graft harvesting on coronal plane alignment and functional outcomes of the lower limb

**DOI:** 10.1038/s41598-024-66847-8

**Published:** 2024-07-09

**Authors:** Abdurrahman Aydın, Mehmet Baydar, Ethem Ayhan Ünkar, Anıl Erbaş, Hanifi Üçpunar, Furkan Yapıcı, Volkan Gür, Kahraman Öztürk

**Affiliations:** 1 Department of Orthopedics and Traumatology, Düzce Akçakoca State Hospital, Cumhuriyet Mh, Park Cd. No: 20, 81650 Akçakoca, Düzce, Turkey; 2 Hand Surgery Clinic, University of Health Sciences Turkey MS Baltalimani Bone Diseases Teaching and Research Hospital, Istanbul, Turkey; 3 Department of Orthopedics and Traumatology, University of Health Sciences Turkey MS Baltalimani Bone Diseases Teaching and Research Hospital, Istanbul, Turkey; 4 Department of Orthopedics and Traumatology, Erzincan Binali Yıldırım University, Erzincan, Turkey

**Keywords:** Vascularized fibula graft, Lower limb length radiograph, Proximal fibular osteotomy, High tibial osteotomy, Musculoskeletal system, Outcomes research

## Abstract

Recent studies on fibular osteotomy for varus gonarthrosis and possible subsequent biomechanical changes have attracted increasing attention to the topic. Existing studies have focused mainly on proximal fibular osteotomy with short follow-up periods. The aim of this study was to investigate changes in the alignment of the coronal plane of the ankle and knee joints in patients who underwent vascularized fibula graft harvest (VFGH). The evaluation was based on functional outcomes and radiological measurements.In the comparison between the VFGH side and the contralateral side, no significant differences in the knee inclination (KI) or talar inclination (TI) angle, knee medial clear space (K-MCS) or ankle medial clear space (A-MCS) distance were noted. However, a significant difference in the hip knee (HKA) angle was observed between the operated and nonoperated sides (0.3° ± 1.8° and 1.5° ± 1.9°, respectively [p = 0.019]). Statistically significant differences in both the knee society score (KSS) and the AOFAS scores were found between the ipsilateral donor limb and the contralateral healthy limb. Although the contralateral healthy side had better clinical scores than the VFGH side, the outcomes of the VFGH side were still satisfactory or excellent.

## Introduction

The fibula is a bone reserve that can be used as an alternative for many surgeries, such as those for pseudoarthrosis, tumors, and hip avascular necrosis (AVN). Fibular grafts can be used as vascularized and non-vascularized. The main factor involved in the preference for vascularized bone grafts over nonvascularized grafts in cases of nonunion and similar complicated cases is insufficient peripheral vascularity in the pathological region. With the continuation of blood flow in vascularized bone grafts, bone union and graft hypertrophy are possible^1^. This also makes the graft more resistant to infection, and faster healing and mechanical loading can be better tolerated due to hypertrophy, thus reducing the incidence of graft resorption and stress fractures^2,3^. The morphology of the fibula, its sufficient length, its biomechanical properties, and the structure of its vascular pedicle make it the first choice for the reconstruction of nonunion in long bones compared to other free bone flaps^4,5^. Especially for bone defects larger than 10 cm, vascularized fibula graft (VFG) transfer should be the optimal method of choice. The preservation of the medullary and periosteal circulation of vascularized bone grafts is important for the treatment of large segmented defects and hip AVNs.

Fibular osteotomies for varus gonarthrosis are becoming increasingly popular^6,7^. This procedure is becoming much more popular in the Eastern world, perhaps becauseit is more straightforward and less expensive and requires less rehabilitation than alternative procedures such as high tibial osteotomy (HTO), unicondyler knee arthroplasty (UKA), and total knee arthroplasty (TKA)^8^. Proximal fibular osteotomy (PFO) aids in the correction of a varus deformity in knee osteoarthritis (KOA), which shifts the loading force from the medial compartment more laterally. Therefore, these procedures help to decrease pain and achieve satisfactory functional recovery. Among them, PFO has gained importance in the current literature^9^.

After PFO, patients are thought to experience alignment changes in the coronal plane, especially in the knee joint. For this reason, it has been claimed that PFO has a role in treating the early stages of knee arthrosis^10,11^.

The mid-to long-term radiological and clinical outcomes of shaft/distal fibula osteotomy/excision or vascularized fibula graft harvest (VFGH) are unclear in the literature. Our study aimed to evaluate the clinical and radiological results of VFGH.

We hypothesized that there would be a valgus change in the HKA angle after VFGH.

## Methods

Patients who underwent VFGH surgery due to femoral head AVN between January 2000 and December 2015 at Metin Sabanci Baltalimani Bone and Joint Diseases Research Hospital (University of Health Sciences,Istanbul, Turkey) were included in the study. Informed consent was obtained from all participants.

This study was authorized by the Institutional Review Board of the University of Health Sciences, Metin Sabanci Baltalimani Bone and Joint Diseases Research Hospital, Istanbul, Turkey (IRB No. 71.513.26.02.2021). All methods in the study were performed in accordance with the relevant guidelines and regulations.

**Figure 1:**
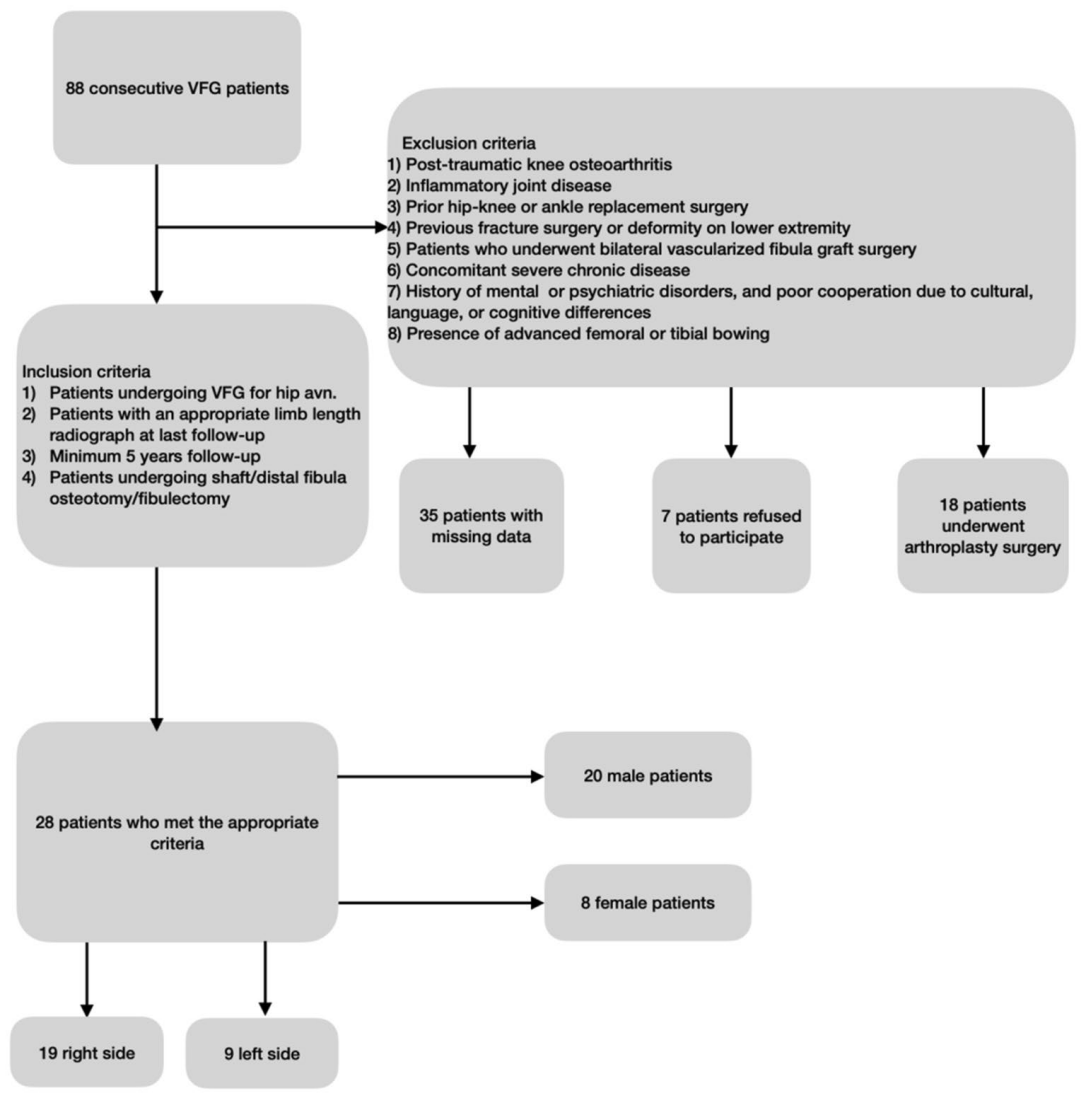
Inclusion and exclusion criteria.

Patients with varus alignment of the HKA, a minimum follow-up period of five years, and a complete series of radiographs were included in the study. The diagnostic criteria for the patients included in the study were shaft/distal fibulectomy, absence of active complaints, regular follow-up and youngactive patients with hip AVN. The exclusion criteria were posttraumatic knee osteoarthritis, inflammatory joint disease, history of previous fracture operation, bilateral fibula grafting, previous deformity correction surgery on the same side that may cause changes in the alignment of the coronal plane, and previous arthroplasty surgery on the same or opposite side (Fig. 1).

The mean follow-up period was 7.8 ± 4.1 years (range 5.3–16.1). Eight patients were female (28%), and 20 were male (72%). The mean age at the first operation was 33.7 ± 9.5 years (range: 21–58). The mean age at the last follow-up was 41.6 ± 10.2 years (range 26–63).

Of the eighty-eight patients, thirty-five were excluded because of missing data, seven because they did not want to participate in the study, and eighteen because they underwent arthroplasty surgery. Nineteen patients underwent surgery on the right side, and nine patients underwent surgery on the left side. All surgeries were performed by the same hand surgery team (team chief, KO), and the data were collected by a single doctor (AA).

### Surgical management

A skin incision was made posterolateral to the fibula, 2 cm longer than the graft length on both sides, and the fibula was exposed between the peroneus and soleus muscle. VFGH was performed by removing the pedicle up to its bifurcation. After harvesting the VFG, the surgical wound was closed with sutures. The leg was covered with a compression bandage.

### Radiographic and clinical evaluation

**Figure 2:**
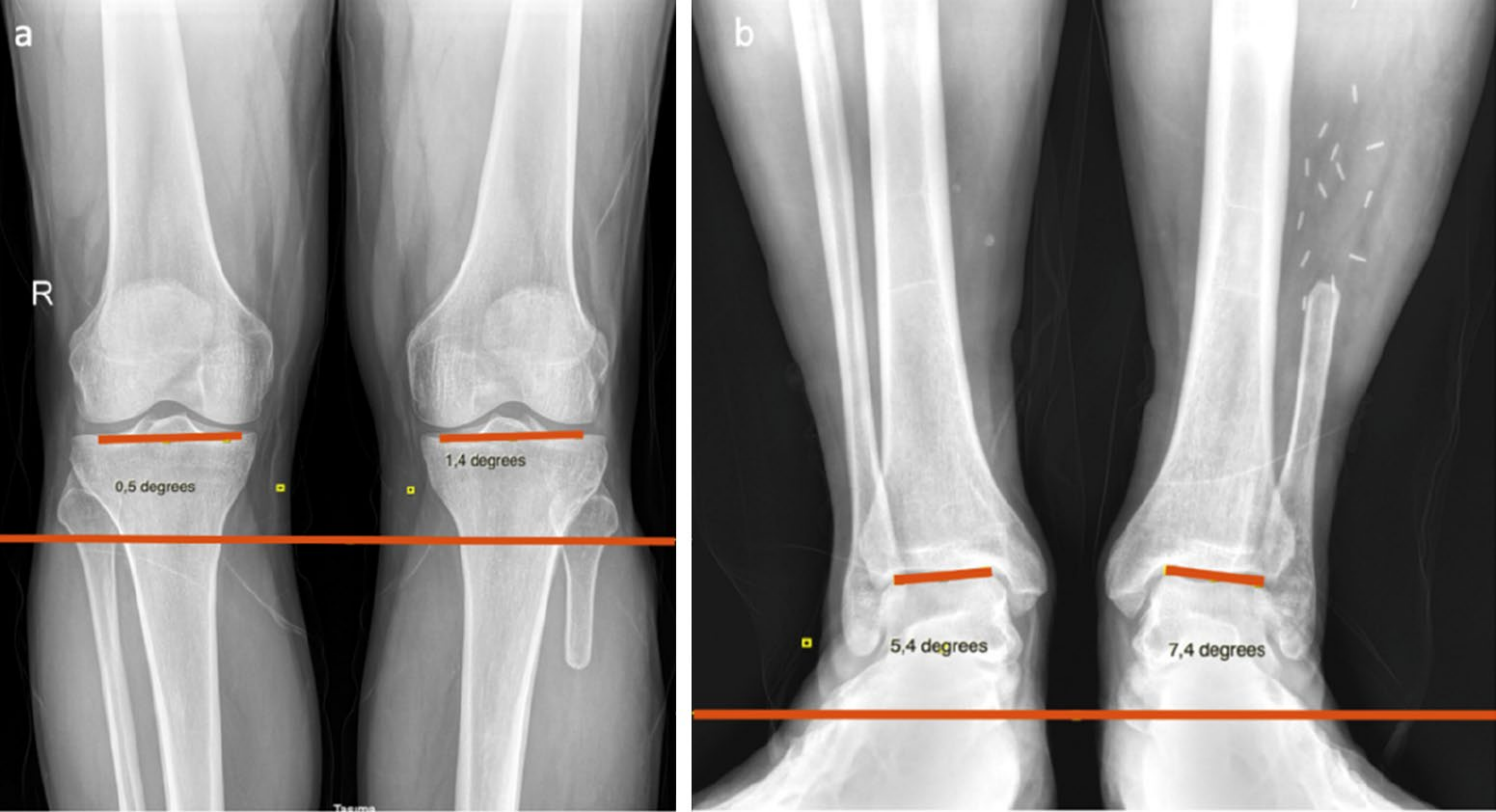
(**a**) KI measurement, (**b**) TI measurement.

**Figure 3:**
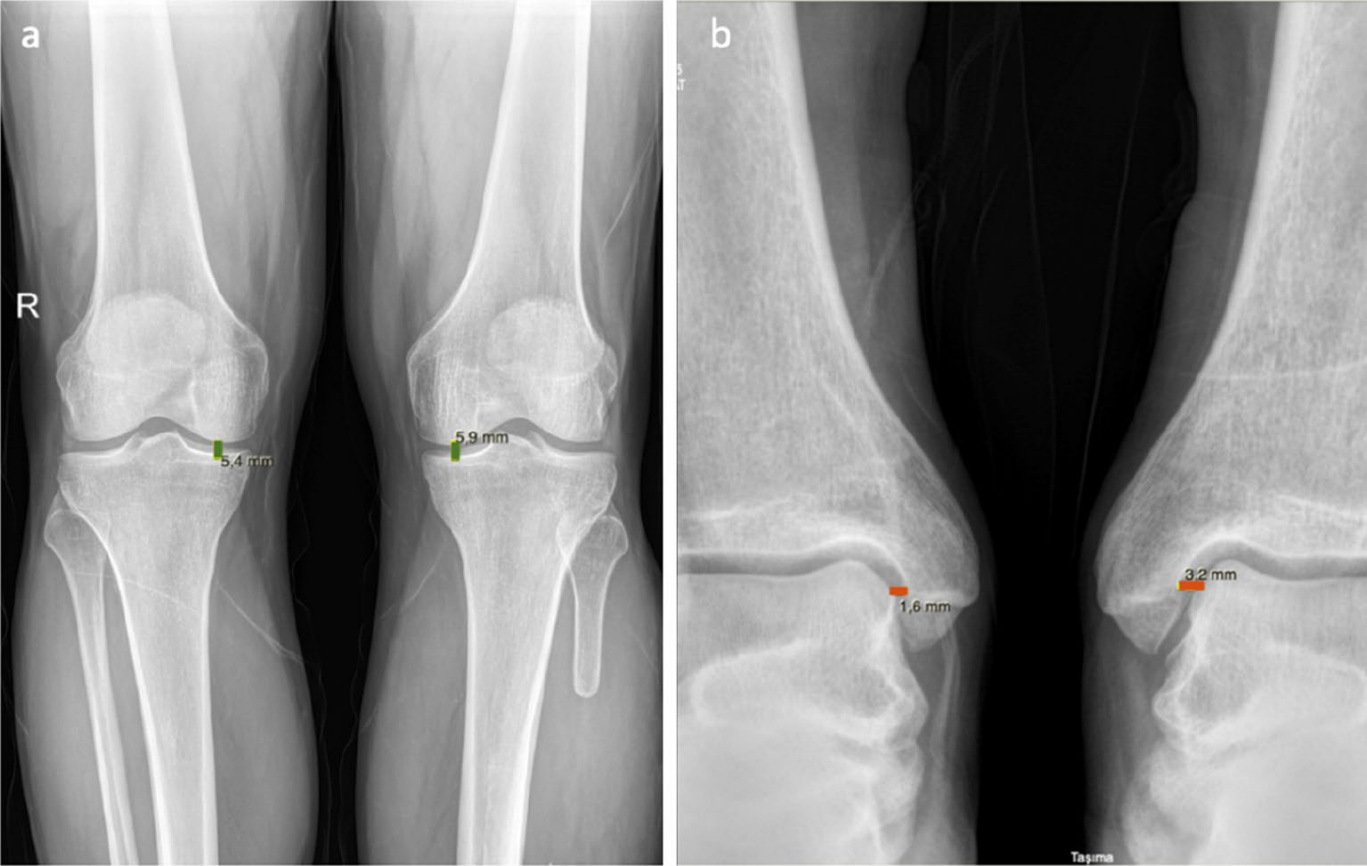
(**a**) measurement of medical joint space width of knee, (**b**) measurement of medial joint space of ankle.

All radiologic parameter measurements of the patients consisted of measurements taken during the last follow-up [7.8 ± 4.1 years (range 5.3–16.1)]. Five measurements were taken to assess the changes in alignment in the coronal plane and three measurements were taken to assess the amount of fibula resected and the amount remaining proximally and distally. Radiologic measurements were performed by an orthopedic surgeon (A.A). Postoperative standing ankle and tibial AP views with both legs bearing weight were taken to assess the hip-knee-ankle angle (HKA), knee-tibial inclination (the acute angle between the perpendicular line and the tibial axis, TI), talar tilt (the acute angle between the horizontal line and the tangent of the superior talar articular surface, TT), medial joint space width of the knee (distance between the apex of the medial condyle of the femur and the posterior end of the tibia), and medial joint space of the ankle (the widest distance between the lateral border of the medial malleolus and the medial side of the talus and is usually measured parallel to the superior talar articular surface)^12–14^. The lengths of the resected fibula and the lengths of the proximal and distal fibula were measured (Figs. 2 and 3).

The American Knee Society Score (KSS) for the knee joint and the Ankle-Hindfoot Scale of the American Orthopedic Foot and Ankle Society (AOFAS) for the ankle joint were calculated and recorded separately for both the operated and contralateral sides during the patient’s last follow-up.

### Rehabilitation and clinical follow-up

After postoperative follow-up, the cleanliness of the dressings was checked on the 2nd postoperative day. After surgery, the patients were wrapped with an elastic bandage, and the dressings of the patients were removed in the 2nd week. In the 3rd month, a partial load was applied to the patients, and a full load was applied in the 6th month. From the 1st postoperative day, the ankle and knee ROMs of the patients were released as much as could be tolerated.

### Statistical analysis

The data analysis was performed using IBM SPSS Statistics for Windows, Version 22.0 (Released 2013; IBM Corp., Armonk, New York, United States), and the data are presented as the mean and standard deviation. Depending on the distribution features of the data, the Mann‒Whitney U test or Student's t test was used to compare all continuous variables. The Kolmogorov‒Smirnov test was used for normality analysis of the data. A pvalue < 0.05 was regarded as significant for all comparisons.

Posthoc power analysis was performed using GPower version 3.1.9.2 (Dusseldorf University, Dusseldorf, Germany). Our study's power was 80.1%, the effect size (d) was 0.8, and the standard error (ɑ) was 0.05.

## Results

At the last follow-up, the mean HKA on the operated side was 0.3° ± 1.8° (range: − 3.3° to 3.7°), whereasthe mean HKA on the contralateral side was 1.5° ± 1.9° (range: − 3.° to 5.7°) (p < 0.05). There was a valgus change in the HKA angles of the patients compared to those on the contralateral side.

All data, including coronal plane measurements of the patients at the last follow-up after surgery, are given in Table 1. Although no significant differences were detected in the knee inclination (KI), knee medical clear space (K-MCS), talar inclination (TI), or anklemedial clear space (A-MCS) measurements (p > 0.05), there was a significant difference in thehip knee ankle (HKA) measurements (p = 0.019). Although the medial joint space increased in the knee and ankle (5.3 ± 1.6 vs. 4.9 ± 1.5 for K-MCS, 3.1 ± 0.7 vs. 2.7 ± 0.9 for A-MCS), no statistically significant difference in the values between the two sides was observed (p > 0.05).

**Table 1:** Comparison of radiological parameters between the operated and nonoperated sides.

	Operated/Nonoperated side	N	Mean	Std. deviation	P value
HKA angle	Operated side	28	0.3	1.8	p = 0.019
	Nonoperated side	28	1.5	1.9	
KI	Operated side	28	1.2	2.1	p > 0.05
	Nonoperated side	28	1.3	2.5	
K-MCS	Operated side	28	5.3	1.6	p > 0.05
	Nonoperated side	28	4.9	1.5	
TI	Operated side	28	0.7	5.1	p > 0.05
	Nonoperated side	28	1.08	5.7	
A-MCS	Operated side	28	3.1	0.7	p > 0.05
	Nonoperated side	28	2.7	0.9	

*HKA* hip knee ankle, *KI* knee inclination, *K-MCS* knee medial clear space, *TI* talar inclination, *A-MCS* ankle medial clear space.

The mean resected fibular length was 16.1 ± 3.3 cm (range 8.9–22.6). The average fibular length proximal to the left was 11.4 ± 4.5 cm (range: 6.1–30.1), whereasthe average fibular length distal to the left was 10.7 ± 3 cm (range: 1.7–17.1). In the comparison of clinical scores, the KSS and AOFAS scores on the operated side were 93.03 ± 10.7 and 90.3 ± 12.8, respectively, whereasthe KSS and AOFAS scores on the contralateral side were 100 and 100, respectively. None of the 28 patients experienced symptomatic knee or ankle pain during the last follow-up. There was a statistically significant difference between the two sides in terms of the KSS and AOFAS scores (p < 0.001) (Table 2). According to the evaluation of the patients' ankle and knee joint ROMs, there was no limitation of movement on the side of the fibula harvested. None of the 28 patients experienced vascularized fibula graft failure, and none of the patients complained of hip pain at the last follow-up. None of the patients required a second surgery during the follow-up period.

**Table 2:** Comparison of clinical scores between the operated and nonoperated sides.

	Operated/Nonoperated side	N	Mean	Std. deviation	P value
KSS score	Operated side	28	93.03	10.7	p < 0.001
	Nonoperated side	28	100		
AOFAS score	Operated side	28	90.03	12.8	p < 0.001
	Nonoperated side	28	100		

*KSS* Knee Society Score, *AOFAS* American Orthopedic Foot & Ankle Society.

## Discussion

Our study demonstrated the results of vascularized fibula graft surgery. In addition, this study revealed that the medium- to long-term results regarding donor site morbidity may be associated with changes in coronal plane alignment in both the knee and ankle. No donor site morbidity was detected in any patient during the last follow-up. Although the clinical scores and functional results at the last follow-up on the operated side were significantly different from those on the contralateral side, they were satisfactory or excellent. All patients continued their lives painlessly and without restrictions at the last follow-up.

The most important result of the study was a mean 1.2° valgus change in the coronal plane alignment of the patients (HKA angle) after VFGH. Nie et al.^15^ reported a change in the HKA angle of 1.24 degrees (from varus to neutral) in patients who underwent proximal partial fibulectomy and found a statistically significant difference compared to the preoperative period. In this study, it was observed that the HKA at 3 and 6 months did not significantly differ. In a study on the clinical and biomechanical results of fibulectomy and drug use in the treatment of medial knee osteoarthritis, it was found that the HKA of patients who underwent proximal fibulectomy changed by 0.99 degrees (from varus to neutral)^16^. In addition, gait analysis was performed in this study, and gait parameters of the patients after proximal fibular osteotomy were evaluated. The lack of gait analysis in our study is a limitation in terms of revealing the biomechanical effect of the study. The absence of active complaints and ROM limitations of the knee and ankle joints at the last follow-up of the patients were also among the important findings. In addition, the healthy side had better clinical scores than did the VFGH side, but the outcomes of the VFGH side were still satisfactory or excellent.

Although there was an increase in the KI and TI, there was no statistically significant difference compared to those values obtained on the contralateral side. Although this change did not create a statistically significant difference, the change they created together resulted in a statistically significant difference between the two sides in the HKA angle. There are a limited number of studies showing changes in the KI and TI after fibular osteotomy. In the study conducted by Choi et al.^17^, it was revealed that HTO produced a change in the valgus direction in both the ankle and knee joints.

Although we observed a mean 1.2° valgus change in the HKA, the clinical importance of this slight change is a matter of debate. Our findings suggested that the contribution of fibular osteotomies to the valgus change in coronal alignment may be overstated. In studies evaluating HTO and PFO in the literature, the change in the HKA angle was less than 5 degrees, and the clinical scores of the patients were significantly improved^18,19^.

Fibular osteotomy seems to be a simple and effective treatment that has gained popularity, especially in patients with varus osteoarthritis, and has been introduced in the current literature^20,21^. In patients with varus osteoarthritis, the medial compartment is compressed, and the medial joint space is narrowed. Fibular osteotomy is considered an effective surgical procedure that can be performed in patients with comorbidities and high surgical risk, patients who cannot undergo TKA or patients with low compliance for HTO^22^. To date, many studies have evaluated the effect of PFO and HTO on the knee joint, but few studies have evaluated the effect on the ankle joint, and no long-term studies have evaluated both the knee joint and the ankle joint together^23,24^. This study focused on the changes in coronal plane alignment that occur in both the ankle and knee joints after fibula grafting and aimed to evaluate the clinical results together with the radiological results.

Although there are many radiological, clinical and cadaveric studies on proximal fibular osteotomy in the literature, there are limited studies on fibular shaft osteotomy^25^. Likewise, these studies mostly focused on changes in the alignment of the coronal plane of either the ankle or knee joint^26^. Proximal osteotomy is particularly likely to produce biomechanical changes due to its close proximity to the tibiofibular joint. There are studies evaluating the change in the HKA after proximal fibular osteotomy, and the change in our study was similar to that reported in the literature (the change in the HKA is less than 5 degrees)^22,27,28^. In our study, changes in the alignment of the coronal plane of both the ankle and knee joints after fibula osteotomy and excision were evaluated. This also shows that a deformity that can be corrected in the knee alone may lead to a deformity in the ankle. Existing studies in the literature have investigated the effect of fibula osteotomy in knees with osteoarthritis^11,19,29^. The aim of our study was to investigate the effect of fibula osteotomy/graft removal on the knee and ankle joints of young healthy patients.

Guo et al.^30^ evaluated the effect of fibula osteotomy on ankle joint orientation and observed changes in the patient's ankle coronal plane alignment after vascularized/nonvascularized fibula excision. In this study, the effect of proximal fibular osteotomy on the ankle joint was evaluated. At the 1-year follow-up, HKA and femorotibial angle (FTA) changes were detected. In addition, no significant differences in the AOFAS or VAS score were observed, although a significant difference was observed in the KSS score after PFO. In our study, a statistically significant difference in the HKA angle was noted,whereas no statistically significant differences in the KI, TI, K-MCS, or A-MCS were observed. The mean follow-up period in this study was 1 year, and the mean follow-up period in our study was 7.8 ± 4.1 years. Another advantage of proximal fibular osteotomy or fibula osteotomy over HTO is that it does not create an obstacle to TKA in cases where total knee arthroplasty is needed. PFO facilitates the correction of deformities in patients with minimal varus deformity but does not provide anatomical valgus alignment. Therefore, this surgery may be recommended for patients with low-grade varus deformity. In patients with more severe deformities, PFO surgery may be recommended in combination with HTO^31^.

There are various studies on fibula biomechanics. Beyond 1/6 load bearing of the fibula, the proximal tibiofibular joint has a complex structure. Current studies have focused on proximal fibula osteotomy. Fibular osteotomies were performed proximally, and the alignment of the knee joint of the patients was evaluated. Guo et al.^30^ reported significant widening of the medial knee joint space at the 1-year follow-up in patients who underwent fibula osteotomy. In a study on the biomechanics of the proximal tibiofibular joint, it was observed that the lateral part of the tibia was more resistant to resistance, and it was observed that the load shifted from medial to lateral due to the broken resistance with osteotomy^5,9^. In our study, osteotomy was performed at an average of 11.4 ± 4.5 cm distal to the fibular head. Our study revealed that middle 1/3 fibular osteotomy did not cause any significant changes in the coronal plane of the ankle or knee joint in the long term. Although there was a mean difference of 1.2° in the valgus direction between the HKA and contralateral sides (p = 0.019), the difference between the two sides in terms of other radiological parameters was not statistically significant.

Although our study revealed a statistically significant change of 1.2° in the HKA, it is important to note that this change is not considered clinically significant because it falls below the threshold of 5 degrees. In a study by Guo et al.^30^, a 2° valgus change in the HKA angle was observedover a 1-year follow-up period, with statistically significant differences also observed in the TT and TI angles in contrast to our findings (p < 0.05). Utomo et al.^7^ followed 15 patients with fourth-degree varus osteoarthritis and reported a 4° change in the HKA angle after PFO, with a significant improvement in symptoms. In a study conducted by Yang et al.^22^ with an average follow-up of 49.1 months, a significant improvement in the KSS and VAS scores of the patients was observed, with a mean change of 3.3 degrees in the HKA angle. In almost all of the studies, the angle change was less than 5 degrees, the severity of pain decreased, and functional improvement was achieved. The present findings imply that the outcome of PFO surgery is not exclusively dependent on the change in the HKA. Femoral abduction and external rotation increase after PFO, and biomechanical changes occur in the proximal tibiofemoral joint. Dynamic changes occur secondary to biomechanical changes and pain decreases^19^. In the study by Huang et al.^32^, proximal osteotomy of the fibula altered the kinematics by increasing femoral external rotation and distal translation of the knee as well as creating a change of approximately 5 degrees in valgus. These changes have been reported to be beneficial for reducing knee pain and increasing early functional recovery^32^.

Based on these studies and our results, we believe that the change in knee alignment (change in the HKA angle) after fibular osteotomy is not the only reason for the relief of pain in the knee. We believe that changes in the proximal tibiofibular joint and the external rotation and abduction of the femur cause dynamic changes in the lower extremity and a decrease in knee pain and contribute to early functional recovery. The well-being of patients can be explained by biomechanical changes and dynamic changes that occur. This shows that the change in the angle of the HKA is not the only factor involved in evaluating the success of PFO surgery.

Extensive studies on donor site morbidity after fibular osteotomy and fibular vascularized/nonvascularized grafts have been performed. Sandoval et al.^33^ reported a complication rate of 9.4%. In the study by Wang et al.^19^, no significant morbidity or limitations were observed after fibular osteotomy. In the study by Sandoval et al.^33^, one of the most common complications after proximal osteotomy was peroneal nerve damage (neuropraxia of the deep branch of the peroneal or fibular nerve), with a rate of 3.4%. In our study, none of the patients developed neurological complications at the last follow-up. Existing fibular osteotomy studies recommend osteotomy from the proximal 6–8 cm. Studies have shown that peroneal nerve palsy is less common after distal osteotomy than after proximal osteotomy^34^. In our study, we performed a middle 1/3 weighted osteotomy. Therefore, we may not have encountered nerve damage. Because the coronal plane may have a similar effect in terms of creating misalignment, performing osteotomies distally will reduce the possibility of nerve interference. This suggests that it is safer to perform distal osteotomy rather than proximal osteotomy to create changes in coronal alignment.

Vascularized/nonvascularized fibula surgery can be used in many surgeries, such as AVN surgery, tumor surgery, pseudoarthrosis surgery and deformity surgery^35^. It constitutes the most basic component of many surgical procedures defined to date. Vascularized fibula surgery is an important joint-sparing surgery, especially after avascular necrosis of the hip. When used with an optimal surgical procedure in the appropriate patient, it preserves the joint structure for many years and provides patients with a secondary chance before hip arthroplasty. In various surgeries, options such as iliac wing grafts, costal grafts, and allografts instead of fibula grafts can be used, and the use of vascularized fibula grafts is indispensable in some surgeries. Many studies have shown that the use of vascularized/nonvascularized fibula grafts does not cause serious morbidity and provides a great advantage in this respect^36^.

Our main hypothesis in this study was that there would be a valgus change in the HKA after VFG. In the comparison of the VFG side with the contralateral side, there was a significant difference in the HKA (mean 1.2° of valgus). The knee and ankle orientation angles were evaluated. Although a difference in terms of KI and TI was noted, no statistically significant difference was found between the two sides.

The limitations of our study include the lack of preoperative orthoroentgenograms in most of the patients and the fact that we evaluated our measurements according to the contralateral side. According to the study of Beckers et al.^37^ that assessed whether the contralateral side can be taken as a reference, the reliability of measurements made according to the contralateral side varies. There is no consensus indicating that the contralateral side is a reliable reference. In our study, fibula osteotomies were performed at a similar level and always at the same point. It is not known whether this approach is superior to osteotomy of other fibular segments. Comparative biomechanical studies evaluating the effects of proximal, shaft or distal osteotomies on the knee joint or ankle joint in similar patients may be enlightening in this regard. In addition, the statistically significant difference in the knee and ankle scores on the operated side compared to those on the contralateral side may be because the patients underwent surgery on that side. This situation constitutes another limitation of the study. Studies with a larger patient population and longer duration in which sagittal plane measurements are also evaluated will guide us better in the future.

## Conclusion

To provide context, VFGH may result in a 1.2° valgus shift in the HKA angle compared to the opposite limb, assuming symmetrical alignment. Since preoperative orthoroentgenograms of the patients were unavailable we used the opposite limb as a reference when assessing changes in patient alignment; therefore, the use of the described technique in lower limb axis correction procedures should be approached with caution, as it is possible that both lower limbs may not be symmetrically aligned. The VFGH patients did not report active complaints or ROM limitations of the knee or ankle joints at the last follow-up. Although the contralateral healthy side had better clinical scores than the VFGH side, the outcomes of the VFGH side were still satisfactory or excellent.

## Data Availability

The datasets generated during and/or analyzed during the current study are available from the corresponding author upon reasonable request.
